# A trivalent protein-based pan-*Betacoronavirus* vaccine elicits cross-neutralizing antibodies against a panel of coronavirus pseudoviruses

**DOI:** 10.1038/s41541-024-00924-x

**Published:** 2024-07-22

**Authors:** Syamala Rani Thimmiraju, Rakesh Adhikari, JeAnna R. Redd, Maria Jose Villar, Jungsoon Lee, Zhuyun Liu, Yi-Lin Chen, Suman Sharma, Amandeep Kaur, Nestor L. Uzcategui, Shannon E. Ronca, Wen-Hsiang Chen, Jason T. Kimata, Bin Zhan, Ulrich Strych, Maria Elena Bottazzi, Peter J. Hotez, Jeroen Pollet

**Affiliations:** 1 Texas Children’s Hospital Center for Vaccine Development, Houston, TX 77030 USA; 2 Department of Pediatrics, National School of Tropical Medicine, Baylor College of Medicine, Houston, TX 77030 USA; 3 Department of Molecular Virology and Microbiology, Baylor College of Medicine, Houston, TX 77030 USA; 4 Department of Biology, Baylor University, Waco, TX 76706 USA; 5 James A. Baker III Institute for Public Policy, Rice University, Houston, TX 77005 USA

**Keywords:** Vaccines, Microbiology

## Abstract

The development of broad-spectrum coronavirus vaccines is essential to prepare for future respiratory virus pandemics. We demonstrated broad neutralization by a trivalent subunit vaccine, formulating the receptor-binding domains of SARS-CoV, MERS-CoV, and SARS-CoV-2 XBB.1.5 with Alum and CpG55.2. Vaccinated mice produced cross-neutralizing antibodies against all three human *Betacoronaviruses* and others currently exclusive to bats, indicating the epitope preservation of the individual antigens during co-formulation and the potential for epitope broadening.

Given the emergence of three highly pathogenic and deadly human *Betacoronaviruses*—severe acute respiratory syndrome (SARS)-CoV, Middle East respiratory syndrome (MERS)-CoV, and SARS-CoV-2— within the past two decades, together with the looming threat of additional zoonotic viral spillovers, creating a broadly protective coronavirus vaccine capable of safeguarding against the five major lineages of the virus (*Embecovirus, Sarbecovirus, Merbecovirus, Nobecovirus*, and *Hibecovirus*)^1^ remains an urgent global health challenge. Compared to current vaccines, which are either species- or variant-specific, a more broadly protective pan-coronavirus vaccine could provide considerable epidemiological, clinical, and economic value to mitigate the global disease burden^2^.

Early during the COVID-19 pandemic, the development of broadly protective coronavirus vaccines was motivated by the observation of cross-clade *Sarbecovirus* neutralizing antibodies in previously SARS-CoV-infected and then Pfizer BioNTech BNT162b2-vaccinated individuals^3^. Now, with support from national and international funding agencies, multiple broadly protective vaccines are under development^4,5^.

We focused our coronavirus vaccine development efforts on low-cost, easy-to-produce RBD-based vaccines^6^. In 2013, we developed RBD219-N1 as a SARS-CoV vaccine that protected in a mouse challenge model^7^, and when MERS-CoV emerged, we produced a MERS RBD vaccine that protected mice against a lethal MERS-CoV infection^8,9^. In 2020, our RBD-based SARS-CoV-2 vaccine was efficacious in various animal models^10,11^ and transitioned into the clinic, where it was proven safe, immunogenic, and effective^12^. With our vaccine technology, two vaccines (Corbevax, Biological E Ltd, India; and Indovac, Bio Farma, Indonesia) were produced for less than $2 per dose and administered well over 100 million times in India and Indonesia, combined. Most recently, Corbevax received an emergency utilization listing (EUL) from the WHO^13^. In addition, Biological E is advancing our latest SARS-CoV-2 vaccine, containing the XBB.1.5 RBD antigen, which successfully cross-neutralizes recent omicron subvariants^14^. The vaccine’s ability to stimulate a lasting humoral immune response was validated in mice for 98 days, as shown in Supplementary Fig. 1, regardless of whether administered as a single dose or a prime/boost regimen. In a phase 1/2 study encompassing 360 subjects, the safety of Corbevax was affirmed for up to 12 months, with notably elevated levels of neutralizing antibody titers persisting for a minimum of 6 months^12^.

Here, we discuss the co-formulation of three CoV RBD antigens alongside Alum and CpG and define the neutralizing efficacy of sera from vaccinated mice against a range of beta and alpha coronaviruses. Seven vaccine formulations were prepared containing all possible combinations of three coronavirus RBD antigens (SARS-CoV, MERS-CoV, and SARS-CoV-2 XBB.1.5) together with Alum and CpG55.2 (kindly provided by Nikolai Petrovsky, Vaxine Pty Ltd, Australia). Groups of twelve mice were immunized intramuscularly twice, three weeks apart. Both the dose and administration regimen were informed by prior studies^7–9,11^. Serum was collected two weeks after the final boost for analysis of antigen-specific IgG, IgG1, and IgG2a titers by ELISA, using immobilized recombinant RBD proteins for SARS-CoV, MERS-CoV, and SARS-CoV-2 XBB.1.5 (Fig. 1). Antibody neutralization was tested against eight pseudoviruses, including *Sarbecoviruses* (SARS-CoV, SARS-CoV-2 XBB.1.5, SARS-CoV-2 JN.1, WIV1, RsSHC014, RaTG13), *Merbecovirus* (MERS-CoV), and a human *Alphacoronavirus* (NL-63) (Fig. 2).

**Fig. 1:**
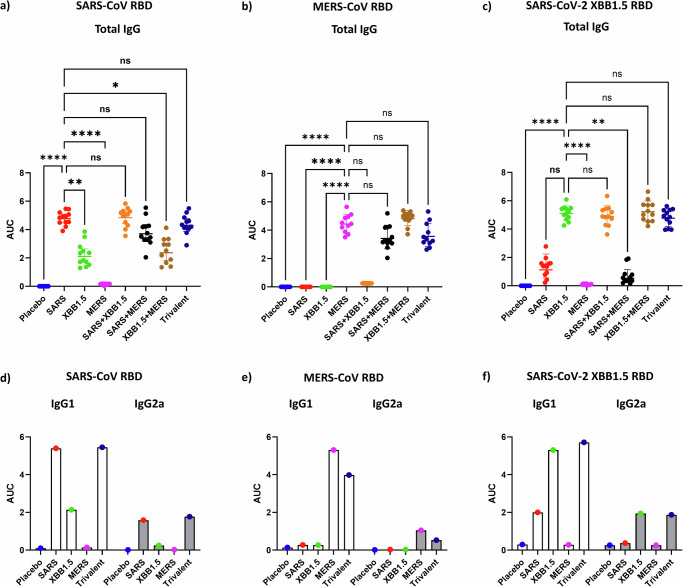
Antigen-specific ELISA. **a**–**c** Total serum IgG levels against recombinant SARS-CoV RBD, MERS-CoV RBD, and SARS-COV XBB1.5 RBD. Each panel’s X-axis represents the different experimental groups. Mice (*n* = 12/group) were immunized twice, 3 weeks apart, with the specified CoV RBDs formulated with Alum + CpG55.2. Serum samples were collected 14 days after the second vaccination and assessed in an ELISA against immobilized, recombinant RBD protein specified in the header of each panel. The Y-axis displays the area under the curve (AUC), derived from the ELISA data of a serum titration curve. Data presenting individual samples alongside geometric means with 95% confidence intervals. Statistical significance for each group against the matching monovalent vaccine group was determined by non-parametric Kruskal–Wallis multiple comparison test *p* > 0.123 (ns), *p* < 0.032 (*), *p* < 0.0021(**), *p* < 0.0001(****). **d**–**f** ELISA AUC of antibody isotypes IgG1 and IgG2a. Sera were pooled for each group, and the data presented is the average of duplicate independent serum dilution series.

**Fig. 2:**
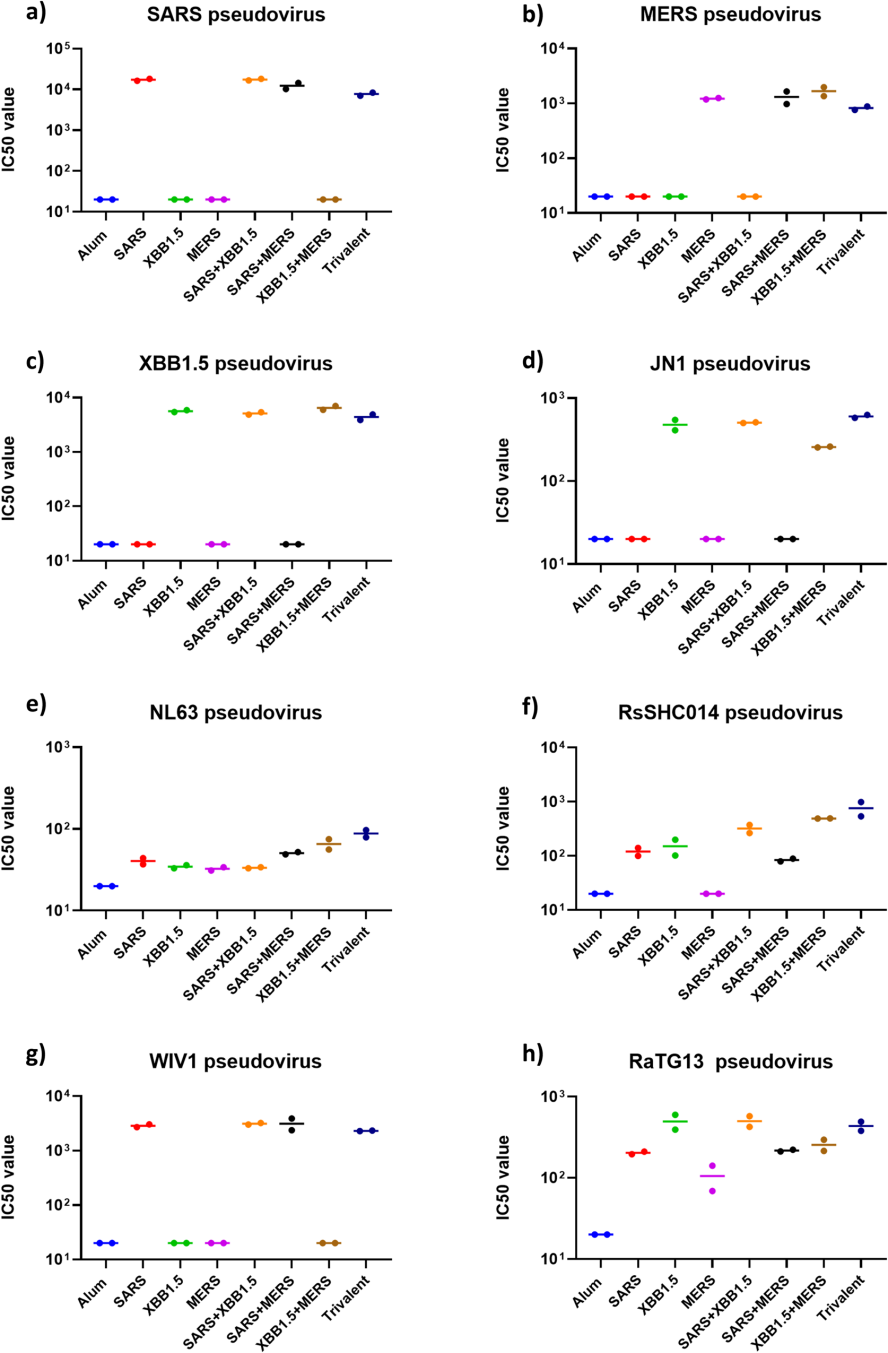
Pan-coronavirus neutralization analysis. Mice (*n* = 12/group) were immunized twice, 3 weeks apart, with the specified CoV RBDs formulated with Alum+CpG55.2. **a**–**h** The neutralization of pan-coronavirus pseudoviruses was assessed using pooled sera for each group. The X-axis denotes the vaccine antigens, while the Y-axis represents neutralizing titers (IC50). Each data point reflects the mean IC50 values obtained from duplicate independent serum dilution series.

Vaccination using monovalent, bivalent, or trivalent combinations of the CoV RBD antigens induced high levels of antigen-specific antibodies (Fig. 1). As expected from their conserved RBD regions (71% amino acid identity), SARS-CoV and SARS-CoV-2 XBB.1.5 antibodies cross-reacted to a high degree, while anti-MERS-CoV RBD antibodies (30 and 29% amino acid identity, respectively) did not cross-react with the *Sarbecovirus* antigens. The serum of mice vaccinated with the trivalent vaccine developed high total antibody titers against all three RBD antigens.

The vaccine-induced RBD-specific antibodies further efficiently neutralized the corresponding pseudoviruses (Fig. 2). Notably, the serum of the mice vaccinated with the trivalent vaccine broadly neutralized all tested betacoronaviruses, including three bat pseudoviruses and the recently emerged JN.1 variant of SARS-CoV-2. Among the nine coronaviruses that are currently known to infect humans, five are presumed to have originated from bats^15^. Therefore, the results of our trivalent vaccine against three bat pseudoviruses may be relevant in mitigating any future pandemics resulting from those bat coronaviruses or the recombined strains of SARS-CoV or SARS-CoV-2. In contrast, the neutralization titers against alphacoronavirus (NL-63) were low. Although the NL-63 RBD is known to bind to ACE2, its three-dimensional structure is different from the other RBDs, and poor cross-neutralization by SARS-CoV or SARS-CoV-2 antibodies has been observed and discussed by others^16^.

Using antigenic cartography^17^, we visualized the relationship between the different pseudoviruses and antigens (Supplementary Fig. 5 and Supplementary Table 1). The three-dimensional map placed the trivalent co-formulated serum in an equidistant central portion to the three monovalent and bivalent sera, indicating a balanced humoral immunogenic response. Our results with the trivalent vaccine demonstrate the concept of cross-neutralization of pseudoviruses across the coronavirus spectrum by combining three antigens. Our data also show minimal epitope suppression for neutralization (as exemplified by no decrease in IC50 titers in trivalent vaccine). These results are decisive for understanding how to mitigate the potential risks posed by the broad spectra of coronaviruses and to further improve the breadth of immune response to develop effective vaccines or therapies against future cross-species spillovers. While we did not investigate the cellular immune response in this study, we had previously conducted a thorough analysis of the cellular immune response with the individual RBD+Alum+CpG formulations, showing a balanced Th1/Th2 cytokine response, with increased levels of anti-inflammatory cytokines IL-4 and IL-10, antiviral interferon IFN-γ, and proinflammatory cytokines IL-6 and IL-2^18^.

## Methods

The preparation of the recombinant RBD proteins from SARS-CoV^7^, MERS-CoV^9^, and SARS-CoV-2^14^ XBB.1.5 has been described previously. The SARS-CoV-2 RBD proteins were prepared with 1xTBS buffer (20 mM of Tris, 100 mM NaCl, and pH 7.5). Before injection, alum and CpG55.2 were added to the protein, and the sample was vortexed for 3 s. Each monovalent vaccine dose contained 7 µg of RBD protein, and the multivalent vaccine formulations contained 7 µg of each RBD antigen. The adjuvant dose remained consistent across all formulations, comprising 200 µg of alum (Alhydrogel®, aluminum hydroxide, Catalog # AJV3012, Croda Inc., Snaith, UK), and 20 µg of CpG55.2 (Vaxine Pty Ltd, Australia). Equal binding of the variant RBD proteins to Alum was verified by Langmuir binding isotherms (Supplementary Fig. 2).

Eight groups of 6–8 weeks old BALB/c mice were vaccinated via the intramuscular route. Prime vaccination was done on day zero, with a boost on day 21. On day 35, the mice were anesthetized with 100 µL of a mixture of ketamine (150 mg/kg) and xylazine (15 mg/kg). Blood was then withdrawn via cardiac puncture, followed by cervical dislocation. Animal experiments were performed in full compliance with the Guide for the Care and Use of Laboratory Animals, 8th edition (National Research Council, 2011), under a protocol (AN-8256) approved by Baylor College of Medicine’s Institutional Animal Care and Use Committee (IACUC). To quantify the humoral immune response, serum antibody ELISAs were performed for antigen-specific IgG, IgG1, and IgG2a. ELISA data (Absorbance at 450 nm) was corrected using each dataset’s average blank values (buffer instead of sera). The ELISA AUC (area under the curve) values from the serum dilutions (Supplementary Figs. 3,4) were calculated using GraphPad Prism 10.

Pseudovirus neutralization assays against SARS-CoV, SARS-CoV-2 XBB.1.5, bCoV WIV1, bCoVRaTG13, bCoV RsSHCO14, and hCoV NL-63 were carried out using HEK-293T cells expressing the human ACE2 receptor. In contrast, assays against MERS-CoV pseudovirus were carried out using HeLa cells expressing the DPP4 receptor.

Coronavirus spike lentiviral-based pseudovirions were generated by transfection of 293T cells as we described previously^14^ using plasmids pNL4-3R-E-luc and pΔ8.9 as the reporter and packaging vectors, respectively, and different spike expression plasmids. pcDNA3.1-XBB.1.5 was used to express the SARS-CoV-2 variant of concern XBB.1.5. Plasmids that express the coronavirus spike proteins for MERS, NL-63, WIV1, and SARS-CoV-1 were obtained from Addgene. pCDNA3.3_MERS_D12 was a gift from David Nemazee (Addgene plasmid # 170448; http://n2t.net/addgene:170448 ; RRID: Addgene_170448)^19^. pcDNA3.3-NL-63-D14 was a gift from David Nemazee (Addgene plasmid # 172666; http://n2t.net/addgene:172666 ; RRID: Addgene_172666). pTwist-WIV1-CoV Δ18 was a gift from Alejandro Balazs (Addgene plasmid # 164439; http://n2t.net/addgene:164439 ; RRID: Addgene_164439)^20,21^. pTwist-SARS-CoV Δ18 was a gift from Alejandro Balazs (Addgene plasmid # 169465; http://n2t.net/addgene:169465 ; RRID: Addgene_169465)^20^.

To express RSsHC014 and RatG13 spikes, we codon-optimized sequences of RSsHC014 and RatG13 spike genes (GenBank: KC881005.1 and MN996532.2). The last 19 codons at the 3′ end were removed to improve incorporation into lentiviral pseudovirions and a flag-tag was added for detection by Western blot. Genscript synthesized and inserted the genes into the pcDNA3.1(+) vector (pcDNA3.1-CoV-RaTG13del19 and pcDNA3.1-CoVRsSHC014del19).

### Generation of a DPP4 expressing Hela cell line

To generate a human DPP4 (CD26) expressing target cell line (Hela-DPP4) for infection with MERS spike pseudovirions, we transduced Hela cells with a DPP4 encoding lentiviral vector, pLEX307-DPP4-puro (pLEX307-DPP4-puro was a gift from Alejandro Chavez & Sho Iketani (Addgene plasmid # 158451; http://n2t.net/addgene:158451; RRID: Addgene_158451)) and selected for vector expressing cells using 1 µg/ml puromycin (Millipore Sigma, P4512) in complete DMEM (high glucose Dulbecco’s modified Eagle’s medium supplemented with 10% heat-inactivated fetal bovine serum, 2 mM l-glutamine, 1 mM sodium pyruvate, 100 U/ml penicillin, 100 µg/ml streptomycin). Puromycin-resistant cells were analyzed for DPP4 (CD26) surface expression by flow cytometry on an Attune Acoustic Focusing Cytometer using a mouse anti-human CD26-PE antibody (clone BA5b, Biolegend, 302705) and were found to be ~90% DPP4 positive. Cells were then live cell sorted on a BD FACSDiscover S8 Cell Sorter for high DPP4 expression, re-analyzed, and shown to be greater than 99% positive for DPP4.

### Supplementary information

Supplementary Information

## Data Availability

The data supporting this study’s findings are available from the corresponding author upon reasonable request.
