# Supplementary material for: A trivalent protein-based pan-Betacoronavirus vaccine elicits cross-neutralizing antibodies against a panel of coronavirus pseudoviruses
Source: NPJ Vaccines. 2024 Jul 22;9:132. doi: 10.1038/s41541-024-00924-x (PMC11271464; doi:10.1038/s41541-024-00924-x)
Supplement: Supplementary file 1 — Supplementary Information [file 41541_2024_924_MOESM1_ESM.pdf]

Supplementary Data

1. Durability of the humoral immune response

Group Label	Group Size	Antigen	Antigen Dose (µg)	Adjuvant Molecule	Adjuvant Dose (µg)	Volume (mL)
1	20	None	-	Alum + CpG	200 + 20	0.05
2	20	RBD203	7	Alum + CpG	200 + 20	0.05
3	20	RBD203	7	Alum + CpG	200 + 20	0.05

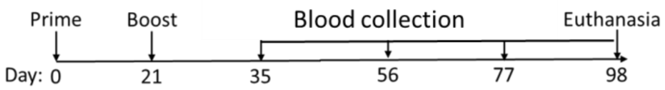

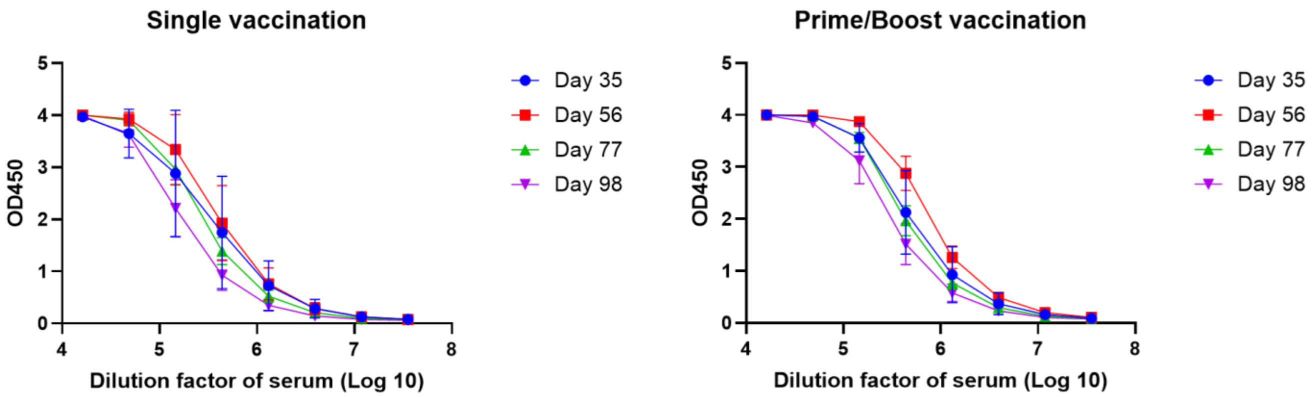

Supplementary Figure 1: Supplementary preclinical immunogenicity study to demonstrate antibody durability. RBD-specific IgG levels in sera from vaccinated Balb/c mice were monitored for up to 98 days. The study comprised three groups: 1. Placebo (Alum + CpG1826 adjuvant only), 2. Single-dose vaccination (7ug SARS-COV-2 RBD/200ug alum/20ug CpG1826), and 3. Prime/boost vaccination administered 21 days apart (7ug SARS-COV-2 RBD/200ug alum/20ug CpG1826). A robust humoral response emerged by day 35, even in mice receiving a single vaccination. Antibody levels exhibited minimal decline over the 98-day period, particularly in mice subjected to two vaccinations. The animal experiment was performed in full compliance with the Guide for the Care and Use of Laboratory Animals, 8th edition (National Research Council, 2011), under a protocol (AN-8256) approved by Baylor College of Medicine's Institutional Animal Care and Use Committee (IACUC).

2. Formulation Study

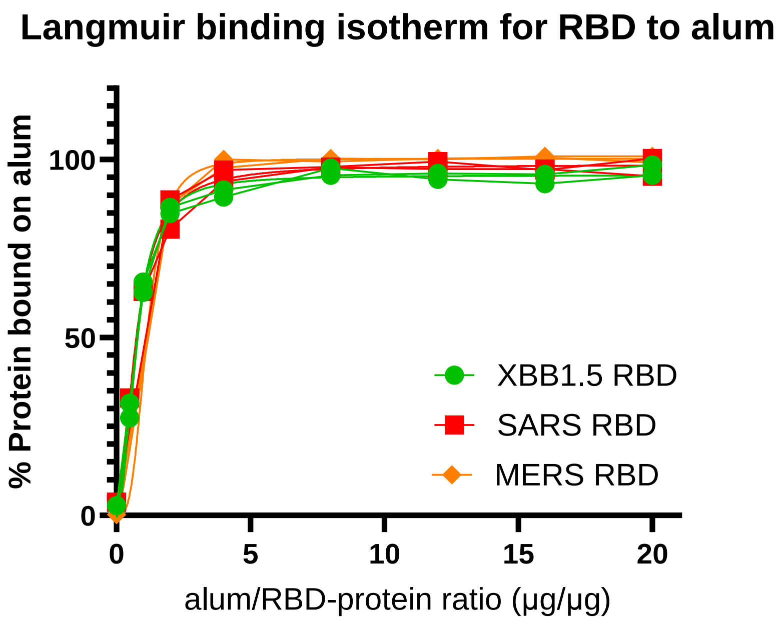

Supplementary Figure 2: Langmuir binding isotherms of coronavirus RBD vaccine antigens to alum. For 8 different weight-based formulation ratios (alum/RBD), the unbound RBD was quantified in the supernatant after spinning down the formulations for 2 minutes. The % protein bound on alum was determined as [100%] - [% protein in supernatant] = [% protein bound on alum]. All three RBD proteins adsorbed strongly to alum when formulated in TBS buffer. The protein adsorption percentage was consistently above 90% when the alum-to-RBD ratio was 8 or higher.

3. Serum ELISA

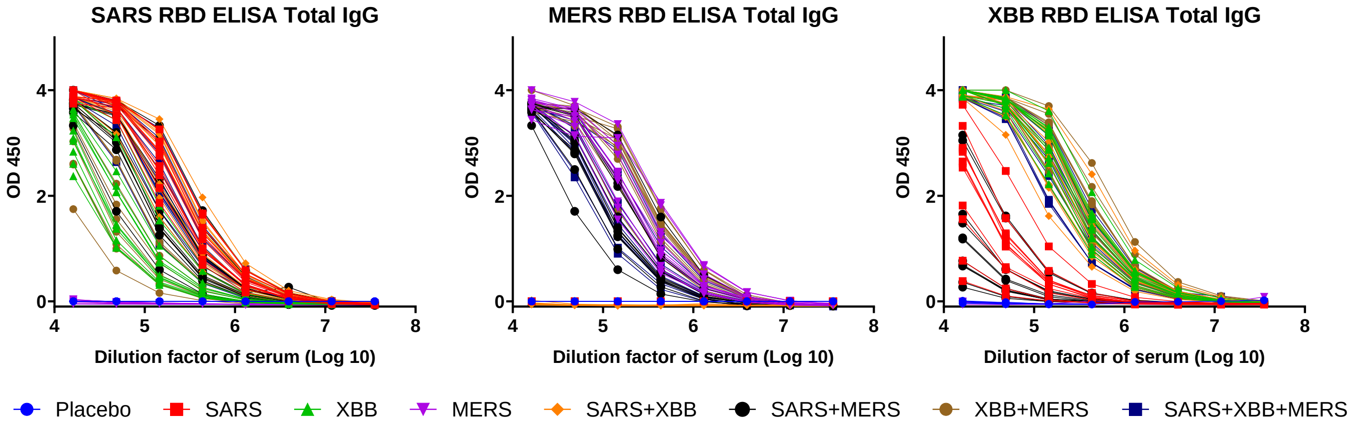

Supplementary Figure 3: Raw Total IgG ELISA Data. ELISA plots of serially diluted sera (n=12 for each vaccine group). Data were corrected using each dataset's average blank value (buffer instead of sera). These plots were used to calculate the Area Under the Curve (AUC) (Main manuscript Figure 1 a-c).

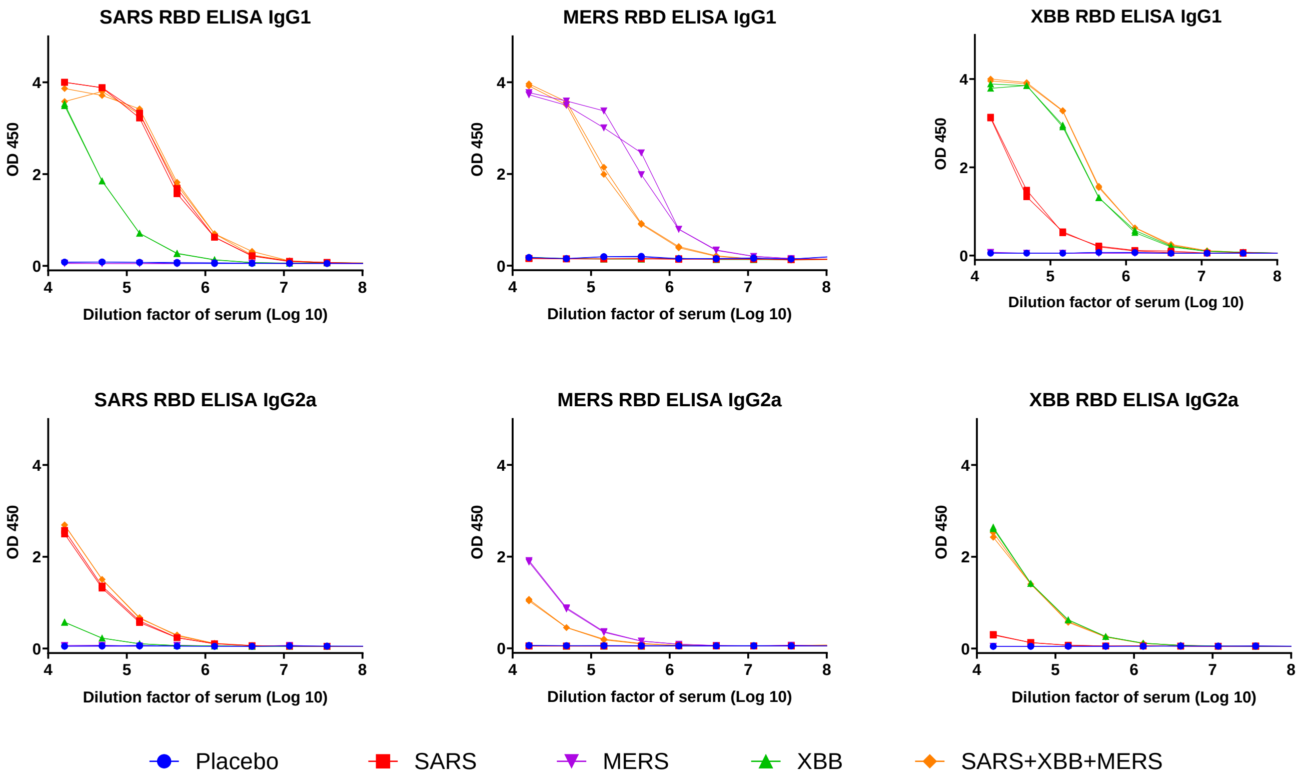

Supplementary Figure 4: Raw IgG1 and IgG2a ELISA data. ELISA plots of serial diluted pooled sera from all mice in each group. Data was collected using duplicates of independent serum dilution series. All data points were corrected using the average blank value (using buffer instead of sera) for each dataset. These plots were used to calculate the Area Under the Curve (AUC) (Main manuscript Figure 1 d-f).

4. Antigenic mapping

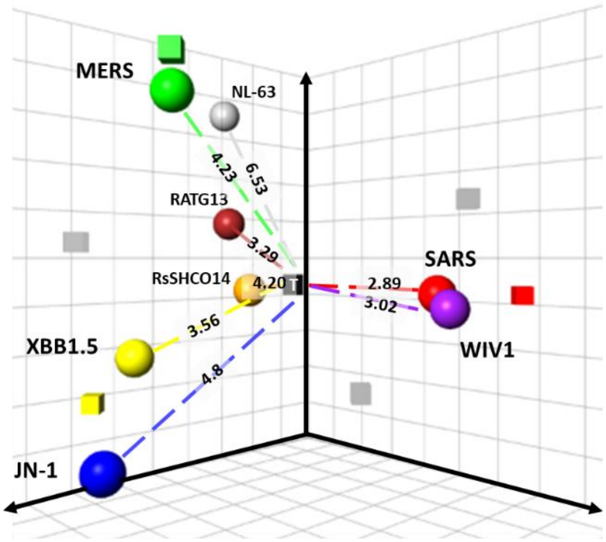

Supplementary Figure 5: Pseudovirus Neutralization Analysis

Three-dimensional antigenic map visualizing virus-serum interactions. Antigenic distances between coronavirus variants were calculated based on serum neutralization IC50 values (R, Racmacs package, version 1.1.4, https://acorg.github.io/Racmacs/). Notably, SARS-CoV-2-like bat viruses cluster towards SARS-Cov-2 XBB 1.5 and JN.1, while MERS and NL-63 pseudoviruses are at a greater distance. In the other plane, the SARS-like bat virus WIV 1 is close proximal to the SARS-CoV pseudovirus. The trivalent co-formulated serum (black box) is centrally positioned equidistant to the three monovalent sera (XBB=yellow, MERS=green, SARS=red) and bivalent sera (grey boxes). The numbers on the map indicate the antigenic distance of trivalent vaccine sera to each pseudovirus variant. Close distances associate with strong neutralization.

Supplementary Table 1: Antigenic distance map (R, Racmacs package, version 1.1.4, https://acorg.github.io/Racmacs/).

		SARS1	XBB1.5	MERS	SARS1+XBB1.5	SARS 1+MERS	XBB 1.5 + MERS	Trivalent
Human-Beta CoVs	MERS PV	8.38	7.01	0.73	6.78	5.82	3.54	4.23
	SARS PV	2.28	7.58	6.83	2.48	2.01	7.08	2.89
	XBB 1.5 PV	8.17	1.80	6.11	4.55	7.26	2.60	3.56
	JN.1 PV	9.14	3.58	7.22	5.10	8.20	4.40	4.80
Human Alpha PV	NL-63 PV	8.61	7.83	6.49	8.26	7.53	6.29	6.53
Bat PVs	WIV 1 PV	2.51	7.74	6.92	2.43	2.13	7.23	3.02
	RSSHCO14 PV	6.98	5.19	6.06	5.60	6.24	4.55	4.20
	RATG13 PV	6.12	4.28	6.54	4.09	5.79	4.56	3.29
